# Hidden Burden of *Bartonella quintana* on the African Continent: Should the Bacterial Infection Be Considered a Neglected Tropical Disease?

**DOI:** 10.1093/ofid/ofad672

**Published:** 2023-12-27

**Authors:** Carl Boodman, Noah Fongwen, Alfonso J Pecoraro, Adane Mihret, Hiwot Abayneh, Pierre-Edouard Fournier, Nitin Gupta, Johan van Griensven

**Affiliations:** Section of Infectious Diseases, Department of Internal Medicine, University of Manitoba, Winnipeg, Manitoba, Canada; Unit of Neglected Tropical Diseases, Clinical Sciences Department, Institute of Tropical Medicine, Antwerp, Belgium; Diagnostics Access, Africa Centres for Disease Control and Prevention, Addis Ababa, Ethiopia; Division of Cardiology, Department of Medicine, Stellenbosch University and Tygerberg Hospital, Cape Town, South Africa; Microbiology Department, Armauer Hansen Research Institute, Addis Ababa, Ethiopia; Microbiology Department, Armauer Hansen Research Institute, Addis Ababa, Ethiopia; French Reference Center for Rickettsioses, Q Fever and Bartonelloses, Institut Hospitalier Universitaire, Marseille, France; Department of Infectious Diseases, Kasturba Medical College, Manipal, India; Unit of Neglected Tropical Diseases, Clinical Sciences Department, Institute of Tropical Medicine, Antwerp, Belgium

**Keywords:** Africa, culture-negative bacterium, infective endocarditis, louse

## Abstract

*Bartonella quintana* is a louse-borne gram-negative bacillus that remains a poorly characterized cause of bacteremia, fever, and infective endocarditis. Due to the link with pediculosis, *B quintana* transmission is tied to poverty, conflict, overcrowding, and inadequate water access to maintain personal hygiene. Although these risk factors may be present globally, we argue that a substantial burden of undocumented *B quintana* infection occurs in Africa due to the high prevalence of these risk factors. Here, we describe the neglected burden of *B quintana* infection, endocarditis, and vector positivity in Africa and evaluate whether *B quintana* meets criteria to be considered a neglected tropical disease according to the World Health Organization.

## Teaser

The presence of *Bartonella quintana* human infection and lice positivity throughout the African continent indicates a hidden burden of illness. Here, we describe how *B quintana* meets criteria to be considered a neglected tropical disease.

## Graphical Abstract

**Graphical Abstract:**
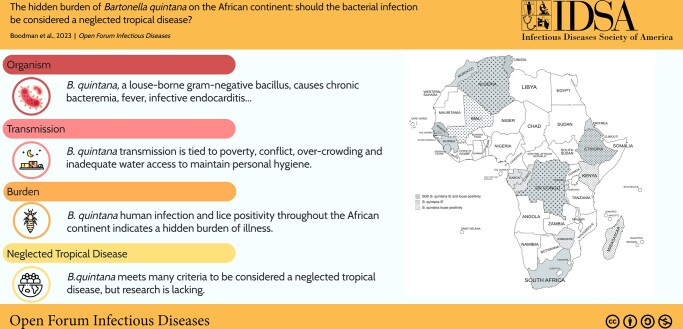

*Bartonella quintana* is a louse-borne gram-negative bacillus that remains a poorly characterized cause of bacteremia, fever, and infective endocarditis (IE) [1]. *B quintana* is primarily transmitted via the inoculation of infected body louse feces into abraded human skin and mucous membranes [1]. Once in the human host, the bacterium infects erythrocytes, causing chronic bacteremia [1]. Due to the link with pediculosis, *B quintana* transmission is tied to poverty, conflict, overcrowding, and inadequate water access to maintain personal hygiene [1, 2]. Although these risk factors may be present globally, we argue that a substantial burden of undocumented *B quintana* infection occurs in Africa due to the high prevalence of these risk factors.

The first description of *B quintana* infection was reported among World War I soldiers, causing a syndrome known as trench fever [3]. In the 1990s, *B quintana* was determined to cause bacteremia and IE among houseless individuals living in cities of high-income countries, leading to the designation “urban trench fever” [1]. While many studies of *B quintana* focus on urban trench fever in Europe and the United States, some of the first documented epidemics of *B quintana* occurred in Africa, with Ethiopian and Tunisian outbreaks occurring in 1946 and 1961, respectively [4–6]. Contemporary descriptions of *B quintana* acquired in Africa are predominantly cases of IE among African inhabitants immigrating to high-income countries outside Africa and diagnosed there.

IE is *B quintana*'s most severe clinical syndrome. *B quintana* IE predominantly affects the aortic valve, causing valvular destruction with large vegetations prone to embolization [1, 7]. Before valvular damage occurs, most cases of *B quintana* IE are associated with months of nonspecific symptoms [1]. Consequently, cases of *B quintana* IE are diagnosed late, after heart failure and embolization occur, if diagnosed at all. Mortality rates due to *B quintana* IE exceed 10%, even with recommended treatment involving antimicrobials and valvular replacement surgery [1]. In 2023, *Bartonella* test positivity was added as a major update to the modified Duke criteria for IE [8].

This update reflects the complexity of diagnosing *Bartonella* infections, including those caused by *B quintana*. Species within the *Bartonella* genus, including *B quintana*, are difficult to culture from blood and are typically not identified via routine 5-day incubation [1]. The bacillus is thus designated a culture-negative bacterial pathogen and a major cause of culture-negative IE (CNIE) [1]. As with the 8 other *Bartonella* species known to cause IE, *B quintana* infection is primarily diagnosed via serologic and molecular techniques [9]. Serology, such as indirect immunofluorescent antibody testing (IFA), identifies current or previous *Bartonella* infection but may be limited by cross-reactivity with other pathogens and by its inability to identify *Bartonella* to the species level, unless cross-adsorption procedures are used, which are restricted to reference laboratories [1, 10]. Serologic positivity of different tests with different pathogen antigens may not exclude possible coexposure to multiple pathogens [11]. Speciating *B quintana* necessitates molecular techniques such as polymerase chain reaction (PCR) with species-specific primers [1, 12]. For cases of IE, sensitivity is highest when testing is performed on explanted cardiac tissue rather than blood samples [1, 9]. The reliance on molecular techniques performed on invasive tissue samples undermines the feasibility of diagnosing *B quintana* in African settings that have limited access to cardiovascular surgery and where 1.3% of the biologic laboratories on the continent perform bacteriologic culture and antimicrobial susceptibility testing [13, 14]. Nevertheless, the number of cases of *B quintana* IE acquired throughout the African continent indicate hidden transmission (Figure 1, Supplementary Appendices 1 and 2). Here, we describe the neglected burden of *B quintana* infection in Africa and evaluate whether *B quintana* meets criteria to be considered a neglected tropical disease (NTD).

**Figure 1.**
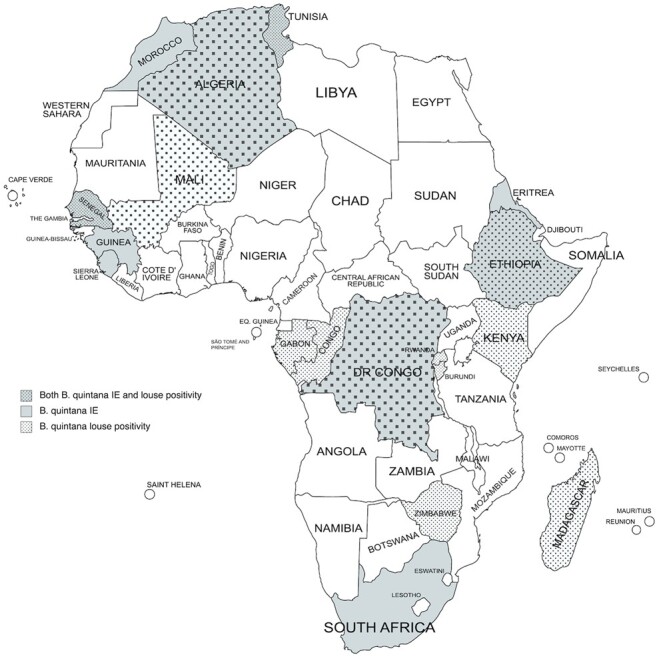
Map of *Bartonella quintana* infective endocarditis and louse positivity on the African continent. References for this map are available in Supplementary Appendices 1 and 2. IE, infective endocarditis.

## *B quintana* in Sub-Saharan Africa

The first documented outbreak of *B quintana* in sub-Saharan Africa occurred in Ethiopia in 1946, and the Horn of Africa continues to be endemic for *B quintana* [4]. In 2017, among 5 Ethiopian children undergoing cardiovascular surgery for IE in Israel, *B quintana* was the main agent in 4 of 5 patients [15]. In 2023, an additional Ethiopian case of *B quintana* endocarditis was added to this series [16]. While the sample size remains small, *B quintana* was the predominant cause of IE in this cohort despite testing for alternate etiologies [15]. In 2021, *B quintana* endocarditis was diagnosed in an Eritrean man living in the Netherlands after presenting with fatigue, renal failure, and weight loss [17]. One year later, an additional case of *B quintana* endocarditis was reported in a 31-year-old Eritrean woman soon after immigrating to Canada [7].

*B quintana* is common in louse vectors in the Horn of Africa. *B quintana* is the most common pathogen in Ethiopian lice, as identified in >9% of 65 head lice pools [18]. While body louse transmission of *B quintana* is well established, entomologic and epidemiologic studies suggest that head lice may occasionally transmit *B quintana,* especially in resource-poor contexts, as highlighted by a recent review article and a *B quintana* outbreak associated with head lice in Senegal [19, 20]. In a study of 271 head and 424 body lice collected from 134 Ethiopian individuals, *B quintana* was identified in 6.7% and 12.7% of those with lice, respectively [21]. *B quintana* positivity in Ethiopian lice and the pathogen's presence among cases of IE acquired in Ethiopia and Eritrea suggest a burden of undiagnosed *B quintana* infection in the Horn of Africa.

Regions of sub-Saharan Africa outside the Horn of Africa have long been known to harbor *B quintana*. Cases of *B quintana* were recognized during a 1995 outbreak of epidemic typhus in Burundi [22]. IE in the region was previously characterized by high rates of CNIE, but recent results from the Tygerberg Endocarditis Cohort suggest that *B quintana* may be the predominant cause CNIE in South Africa. The Tygerberg Endocarditis Cohort is a study of patients with endocarditis treated at Tygerberg Academic Hospital, Cape Town, South Africa [23]. This study included 140 patients, of which 65 were recruited prospectively. In the prospective arm, patients with suspected IE were subjected to a set protocol for organism detection, including serology for *Bartonella* species and routine tissue PCR at the time of surgery [23]. This study identified *B quintana* as the most common cause of CNIE and the second-most common cause of IE after *Staphylococcus aureus* [23]. This finding supports the notion that *B quintana* may be a major contributor to the high rates of CNIE in Southern Africa.

In 2022, an outbreak of febrile illness due to *B quintana* occurred in the Senegalese village of Ndiop [20]. Of the 228 patients whose blood sample was tested for *B quintana*, 4.4% (n = 10) were positive by real-time PCR [20]. Previously, 3 cases of *B quintana* IE acquired in Senegal were diagnosed in Europe, including a pediatric case of a 13-year-old girl and a fatal case of a 50-year-old man [24, 25]. In 2019, *B quintana* IE was reported in a 37-year-old school teacher after immigrating from the Democratic Republic of Congo to the United States [26]. Here too, the authors maintained that the infection was acquired in the Democratic Republic of Congo. In 2023, there was a case of *B quintana* IE in an 11-year-old patient from Rwanda who underwent valve replacement surgery in Israel [16]. These descriptions exemplify cases of *B quintana* endocarditis acquired in Africa but diagnosed and treated outside the African continent.

The sub-Saharan African presence of infection due to *Bartonella* species and *B quintana* was demonstrated in a 2021 multicenter study of febrile illness in African children: *Bartonella* species were among the first- and second-most common origins of bacterial DNA found in the blood of patients in Madagascar and Burkina Faso who were febrile, as identified in 2.5% and 2% of all acute fever cases, respectively [27]. Of the 9 *Bartonella* cases from Madagascar, 4 were speciated to *B quintana* [27]. No other *Bartonella* species were identified [27]. While this study did not identify all *Bartonella-*associated febrile illness to the species level, the only *Bartonella* cases that underwent sequencing were identified as *B quintana*, supporting the notion that *Bartonella* species—and *B quintana* specifically—likely remain common and underrecognized causes of fever in sub-Saharan Africa.

## *B quintana* in North Africa

Many of the first cases of *B quintana* IE were acquired in North Africa, and recent cohorts from the region identify *B quintana* as a common cause of CNIE. The first African case of *B quintana* IE was reported in 1996 in a 34-year-old Algerian farmer, only 3 years after the first publication on *B quintana* IE in 1993 [5, 28]. As the Algerian farmer denied houselessness, alcohol use, and ectoparasitosis, this description provided the first suggestion that risk factors for *B quintana* “may be different in western Europe or North America and in Africa” [28]. Subsequently, 14 other Algerian cases of *B quintana* IE were described, and 12 cases were identified from Tunisia, leading the authors to conclude that the disease “seems to be very common in Tunisia” [29, 30]. While specific risk factors for *B quintana* infection in North Africa have not been explicitly studied, the majority of *B quintana* IE from Algeria and Tunisia was among individuals with “poor living conditions” who lived in crowded households “of at least 10 persons” [29, 30]. This suggests that overcrowding and poverty are risk factors for *B quintana* infection, independent of houselessness and alcohol use disorder.

## Estimating the Hidden Burden of *B quintana* in Africa

The available evidence on human *B quintana* infection acquired in Africa is disproportionately based on cases of IE treated in high-income countries outside Africa and regions within Africa with access to cardiovascular services. This reflects bias due to the unequal availability of cardiovascular surgery and molecular diagnostics [14, 31]. Estimating the burden of *B quintana* in Africa is limited by the compound difficulties of identifying *B quintana* infection microbiologically and diagnosing endocarditis clinically. Estimating *B quintana* prevalence is problematic as those who are infected may have subclinical disease and may not seek care. These difficulties are exacerbated by the fact that the disease is not notifiable.

Studies of houseless populations in high-income countries suggest that >15% of individuals had prior *B quintana* infection and 5% to 14% had *B quintana* bacteremia [1, 32]. Applying these figures to African jurisdictions where *B quintana* IE has been reported would suggest a burden of undiagnosed infection, even if the risk would be several fold lower in this context. Considering that 20% of patients with *B quintana* bacteremia may develop IE, a substantial number of *B quintana* IE may be overlooked [1, 33]. This issue is complicated by the chronicity of *B quintana* bacteremia, which may persist well over a year despite minimal symptoms [6, 34].

While large studies of heart failure and IE in Africa are scant, the existing data suggest a hidden burden of CNIE, possibly consistent with *B quintana* [14, 31]. In a cross-sectional study of 106 Ethiopian children admitted with acute heart failure, IE was the most common cardiac etiology [35]. In a systematic review of IE in Africa, half of the cases were culture negative [14, 23]. While direct evidence of *B quintana* IE in large prospective African studies is limited to the Tygerberg Endocarditis Cohort and the studies described here, it is possible that many of the cases of CNIE are due to undiagnosed *B quintana*. This systematic review also characterizes IE in Africa as a disease of young patients, a finding reflected in the cases of *B quintana* IE described earlier [14].

## Does *B quintana* Meet Criteria to Be Considered a Neglected Tropical Disease?

NTDs are a diverse grouping of diseases united by their association with poverty and their disproportionate burden in tropical and subtropical areas [36]. The World Health Organization (WHO) has 4 criteria for designating a condition as an NTD (Table 1) [36]. Existing evidence suggests that *B quintana* may meet these criteria, though additional research is needed.

**Table 1.** WHO Criteria for Classifying a Condition as a Neglected Tropical Disease and Applicability to *Bartonella quintana*

WHO Criteria for Neglected Tropical Disease Classification	Applicability to *B quintana*
1. Disproportionately affect populations living in poverty and cause important morbidity and mortality—including stigma and discrimination.	*B quintana* infection is associated with conditions of severe deprivation (eg, houselessness, living in a refugee camp, lacking running water to maintain hygiene). *B quintana* endocarditis causes morbidity (heart failure, renal dysfunction, cerebral embolization causing neurologic impairment) and mortality (>10% despite treatment). Pediculosis associated with stigma.
2. Primarily affects populations living in tropical and subtropical areas.	Europe-Africa gradient with greater burden in Africa. Reliance on molecular testing and cardiovascular surgery creates bias against reporting in low- and middle-income countries.
3. Is immediately amenable to broad control, elimination, or eradication by applying ≥1 of the 5 public health strategies adopted by the Department for Control of Neglected Tropical Diseases.	*B quintana* infection may be treated with preventable chemotherapy (eg, doxycycline). Intensified case management may prevent ongoing household transmission (eg, via shared bedding). Vector control: treatment of pediculosis (eg, washing clothing and bedding, pediculicidal therapy with permethrin or ivermectin). Veterinary public health: not applicable. Primarily human pathogen. Infrequent evidence of cat and monkey infection. Safe water, sanitation, and hygiene: access to water to maintain hygiene is essential to interrupt transmission.
4. Is relatively neglected by research—specifically, resource allocation is not commensurate with the magnitude of the problem.	Epidemiology: not notifiable disease. Hidden burden. No prevalence data. No screening guidelines. Diagnostics: no new development of diagnostics. No rapid diagnostic tests. Current diagnostics (eg, immunofluorescent antibody testing, quantitative polymerase chain reaction) not available in many low-resource settings. Treatment: Antimicrobial therapy based on limited evidence (single open trial of gentamicin and doxycycline vs placebo). Toxicity associated with gentamicin. Elevated mortality despite treatment. Anecdotal reports of success with other regimens.

Abbreviation: WHO, World Health Organization.

## *B quintana* Disproportionately Affects Populations Experiencing Poverty

*B quintana* causes morbidity and mortality among populations experiencing houselessness, displacement, and lack of access to running water to maintain personal hygiene [1, 2]. Within Africa, *B quintana* positivity in lice is correlated to country gross domestic product [2]. Countries with lower gross domestic product have a higher prevalence of lice with *B quintana*, though specific risk factors for *B quintana* infection in the African context have not been thoroughly investigated [2]. *B quintana* IE causes morbidity in the form of heart failure, renal dysfunction, and embolization [1]. As access to cardiovascular surgery is limited in many African contexts, mortality due to *B quintana* IE may be simultaneously amplified and underreported. Stigma occurs via the association with ectoparasitosis. Individuals with pediculosis may be shunned due to fears of transmission, pruritis, and the development of skin abrasions. As illustrated by the cases presented here, *B quintana* acquired in Africa disproportionately affects a younger population, for an additional economic burden on families, although cohort studies that incorporate an economic analysis are needed to better elucidate the cost of *B quintana* infection in Africa.

## *B quintana* Primarily Affects Populations Living in Tropical and Subtropical Areas

While *B quintana* was historically described as being localized to Northern Europe, the disproportionate burden of *B quintana* infection in Africa has long been postulated, including a gradient of increasing *B quintana* infection from Northern Europe to African countries [29]. This likely reflects an economic gradient rather than a climactic one [2]. The preponderance of *B quintana* cases reported from high-income countries in nontropical areas likely reflects the combined availability of cardiovascular surgery and molecular diagnostics to speciate *B quintana*, rather than a large burden of disease, as indicated by the cases of *B quintana* endocarditis from northern high-income countries where the pathogen was determined to be acquired in Africa [26, 37–40]. The largest description of *Bartonella* endocarditis is a French study where *B quintana* was detected in 48 (53%) cases [39]. A recent systematic review of *B quintana* endocarditis identified 105 (62.9%) cases to be acquired in a high-income country [40]. More studies are needed to determine the true burden of *B quintana* infection in low- and middle-income countries, including those on the African continent.

## *B quintana* Is Immediately Amenable to Broad Control

*B quintana* infection may be eliminated by applying a combination of 4 of the 5 public health strategies adopted by the WHO for control of NTDs [36]. Oral antimicrobials, such as doxycycline, may be used as chemotherapy to prevent *B quintana* disease in areas with elevated *B quintana* infection, as occurs with mass drug administration for other neglected infections, though studies involving *B quintana* are lacking [41]. Intensified case management and household contract tracing may use existing technology, such as IFA, to screen people for infection prior to endocarditis-related morbidity and mortality. Vector control is feasible by using existing pediculicidal agents, such as permethrin and ivermectin, and interventions to improve access to water to maintain personal hygiene. The latter may be integrated into existing WASH interventions (water, sanitation, and hygiene) that are known to be indispensable components of NTD control.

## Research on *B quintana* Is Neglected

All aspects of *B quintana* research are neglected. *B quintana* epidemiology is poorly described, with most studies focusing on houseless populations in high-income countries, as exemplified by studies of *B quintana* bacteremia among houseless persons in the United States and France [1, 5, 6, 42]. Seroprevalence studies in Africa are lacking. No large studies describe the prevalence of *B quintana* infection among people living on the African continent and presenting with fever, heart failure, or symptoms of embolization. Diagnosis of *B quintana* is complex, relying on expensive equipment and specialized personnel. IFA, the main serologic test for *B quintana*, has low throughput and necessitates a fluorescent microscope, which may not be available in laboratories in certain low-resource settings. Interpretation of IFA requires specific training. Many of the diagnostic companies that produce commercial *B quintana* IFA kits do not distribute to certain African countries where *B quintana* is endemic [18]. Isolating *B quintana* from blood culture samples necessitates specific techniques, such as lysis centrifugation, freeze and thaw, subculturing, and prolonged incubation up to 45 days [1, 43]. If growth occurs, the pathogen is excluded from many commercial databases for interpreting results based on matrix-assisted laser desorption and ionization time-of-flight mass spectrometry, requiring the inclusion of additional spectra limited to specific international reference laboratories [44, 45]. PCR from whole blood has limited sensitivity as compared with its performance on invasive tissue samples, and there is no consensus on the best molecular target to use [46, 47]. Even in high-income countries, *B quintana* testing is often centralized in reference laboratories. *B quintana* serology and PCR are not available in most African countries, despite the capacity of many national laboratories to perform these tests. Rapid diagnostic tests, such as immunochromatographic tests, do not exist, and none are in development. Treatment for *B quintana* is largely based on a small open trial of gentamicin and doxycycline vs placebo for treatment of chronic bacteremia [48]. As gentamicin is associated with significant toxicity, drug-level monitoring is recommended but is rarely available in low-resource settings where the infection is endemic. American resources recommend treatment with rifampin and doxycycline based on limited data [49]. There are no published or registered randomized controlled trials comparing the efficacy of different antimicrobial regimens.

## Public Health Recommendations

While *B quintana* may meet all 4 WHO NTD criteria to be considered for elimination, the current state of research on *B quintana* is so severely deficient that further research is needed to confidently define the condition as an NTD (Table 2). We believe that a few modest measures may substantially improve awareness of this neglected disease. We advocate for *B quintana* to be considered a national notifiable disease to ensure that data on existing cases are reported. We suggest making *B quintana* testing available at national reference laboratories capable of performing serology and PCR, as this would use existing infrastructure and trained personnel to avoid significant financial costs. We suggest that initial surveillance testing occur in batch among patients at high risk with an elevated pretest probability of *B quintana* infection, such as those with CNIE. For countries without capacity to perform *B quintana* testing, we propose that samples be sent to regional reference laboratories with dedicated *B quintana* diagnostic capacity, as occurs with other infections [50]. We encourage the development of immunochromatographic tests to facilitate surveillance in low-resource settings. Last, we advocate for randomized controlled trials to identify safer alternatives to gentamicin. Early detection and treatment of subclinical *B quintana* infection may prevent avoidable morbidity, mortality, and cost.

**Table 2.** Key *Bartonella quintana* Knowledge Gaps and Associated WHO Criteria for Classifying a Condition as a Neglected Tropical Disease

WHO Criteria for Neglected Tropical Disease Classification	Key Knowledge Gaps
1. Disproportionately affect populations living in poverty and cause important morbidity and mortality—including stigma and discrimination.	Risk factors for *B quintana* infection in LMICs are poorly defined and largely extrapolated from studies in high-income countries. Case-control studies in LMICs are lacking. Morbidity and mortality due to *B quintana* infection and endocarditis are poorly characterized in LMICs. Long-term cohort studies of *B quintana* infection and infective endocarditis are lacking, as well as prevalence studies among individuals with heart failure and embolization such as stroke. Qualitative research on pediculosis-related stigma and discrimination is absent. The effect of ectoparasitosis on employment, social mobility, and marriageability is not well studied.
2. Primarily affects populations living in tropical and subtropical areas.	While a Europe-Africa gradient has been proposed, comparative prevalence studies among countries do not exist. With few exceptions (eg, Tygerberg Endocarditis Cohort–South Africa), existing data predominantly rely on diagnostics performed outside LMICs, reflecting the need for diagnostic capacity in referral laboratories in LMICs.
3. Is immediately amenable to broad control, elimination, or eradication by applying ≥1 of the 5 public health strategies adopted by the Department for Control of Neglected Tropical Diseases.	Studies evaluating the role of oral chemotherapy to prevent the progression from infection to endovascular disease are lacking. Environmental stability of *B quintana* on shared bedding/clothing and its role in transmission is poorly defined. Transmission in LMICs is poorly characterized. Many case reports of patients with *B quintana* infective endocarditis acquired in LMICs deny previous pediculosis, suggesting alternate forms of transmission. Case-control, contact tracing, and additional vector studies are required.
4. Is relatively neglected by research—specifically, resource allocation is not commensurate with the magnitude of the problem.	Little to no investment in most aspects of *Bartonella* research—The Steven & Alexandra Cohen Foundation being a recent exception. *Bartonella* research needs, stakeholders, and existing projects have not been mapped.

Abbreviations: LMICs, low- and middle-income countries; WHO, World Health Organization.

The inverse care law states that the availability of care “tends to vary inversely with the need of the population served” [51]. *B quintana* predominantly affects individuals experiencing substantial need, with a concealed burden on the African continent. Recognizing that *B quintana* meets many NTD criteria is a first necessary step.

## Supplementary Material

ofad672_Supplementary_Data

## Supplementary Data

Supplementary materials are available at *Open Forum Infectious Diseases* online. Consisting of data provided by the authors to benefit the reader, the posted materials are not copyedited and are the sole responsibility of the authors, so questions or comments should be addressed to the corresponding author.

## Notes

***Acknowledgments.*** C. B.’s salary is supported by the University of Manitoba's Clinical Investigator Program. No other funding source is associated with this publication.

***Author contributions***. C. B.: conceptualization, methodology, investigation, writing (original draft), project administration. N. F.: writing (review and editing). A. J. P.: writing (review and editing), supervision. A. M.: writing (review and editing), methodology, resources/investigation. H. A.: writing (review and editing), investigation. P.-E. F.: writing (review and editing), supervision. N. G.: writing (review and editing), project administration. J. v. G: conceptualization, writing (review and editing), supervision.

***Patient consent statement***: This study exclusively uses existing published data and thus does not include factors necessitating patient consent or ethical committee approval.

## References

[1] Foucault C , Brouqui P, Raoult D. *Bartonella quintana* characteristics and clinical management. Emerg Infect Dis J 2006; 12:217–23. 10.3201/eid1202.050874 PMC3373112 16494745

[2] Sangaré AK , Boutellis A, Drali R, et al Detection of *Bartonella quintana* in African body and head lice. Am Soc Trop Med Hyg 2014; 91:294–301. 10.4269/ajtmh.13-0707 PMC4125252 24935950

[3] Graham JHP . A note on a relapsing febrile illness of unknown origin. Lancet 1915; 186:703–4.

[4] Vinson JW . In vitro cultivation of the rickettsial agent of trench fever. Bull World Health Organ 1966; 35:155–64. 5297000 PMC2476134

[5] Ohl ME , Spach DH. *Bartonella quintana* and urban trench fever. Clin Infect Dis 2000; 31:131–5. 10913410 10.1086/313890

[6] Foucault C , Barrau K, Brouqui P, Raoult D. *Bartonella quintana* bacteremia among homeless people. Clin Infect Dis 2002; 35:684–9. 12203165 10.1086/342065

[7] Boodman C , Wuerz T, Lagacé-Wiens P, et al Serologic testing for *Bartonella* in Manitoba, Canada, 2010–2020: a retrospective case series. CMAJ Open 2022; 10:E476–82. 10.9778/cmajo.20210180 PMC9177198 35640989

[8] Fowler VG Jr , Durack DT, Selton-Suty C, et al The 2023 Duke-ISCVID criteria for infective endocarditis: updating the modified Duke criteria. Clin Infect Dis 2023; 77:518–26.37138445

[9] Okaro U , Addisu A, Casanas B, Anderson B. *Bartonella* species, an emerging cause of blood-culture-negative endocarditis. Clin Microbiol Rev 2017; 30:709–46. 28490579 10.1128/CMR.00013-17 PMC5475225

[10] Boodman C , Gupta N. Schrödinger's cat paradox: *Bartonella* serology cannot be used to speciate *Bartonella* endocarditis. Open Forum Infect Dis 2023; 10:ofad436. 37663087 10.1093/ofid/ofad436 PMC10468726

[11] Thiel N , Baker M, Lipton B, Fuller L, Breitschwerdt EB, Rabinowitz P. Risk factors for *Bartonella* seroreactivity among veterinary workers in the Pacific Northwest. Vector Borne Zoonotic Dis 2023; 23:356–63. 37326985 10.1089/vbz.2022.0060 PMC10398744

[12] McCormick D , Rassoulian-Barrett S, Hoogestraat D, et al *Bartonella* spp infections identified by molecular methods, United States. Emerg Infect Dis J 2023; 29:467–76. 10.3201/eid2903.221223 PMC9973681 36823096

[13] African Society for Laboratory Medicine . Mapping Antimicrobial Resistance and Antimicrobial Use Partnership. 2023. Available at: https://aslm.org/what-we-do/maap/. Accessed 2 October 2023.

[14] Noubiap JJ , Nkeck JR, Kwondom BS, Nyaga UF. Epidemiology of infective endocarditis in Africa: a systematic review and meta-analysis. Lancet Glob Health 2022; 10:e77–86. 34919859 10.1016/S2214-109X(21)00400-9

[15] Tasher D , Raucher-Sternfeld A, Tamir A, Giladi M, Somekh E. *Bartonella quintana*, an unrecognized cause of infective endocarditis in children in Ethiopia. Emerg Infect Dis 2017; 23:1246–52. 28730981 10.3201/eid2308.161037 PMC5547792

[16] Sato S , Shapira L, Tasher D, Maruyama S, Giladi M. Molecular epidemiology of *Bartonella quintana* endocarditis in patients from Israel and Eastern Africa. BMC Infect Dis 2023; 23:142. 36882746 10.1186/s12879-023-08099-x PMC9993625

[17] Plantinga NL , Vos RJ, Georgieva L, Roescher N. *Bartonella quintana* as a cause for prosthetic valve endocarditis and post-sternotomy mediastinitis. Access Microbiol 2021; 3:000217. 34151169 10.1099/acmi.0.000217 PMC8209714

[18] Cutler S , Abdissa A, Adamu H, Tolosa T, Gashaw A. *Bartonella quintana* in Ethiopian lice. Comp Immunol Microbiol Infect Dis 2012; 35:17–21. 22019400 10.1016/j.cimid.2011.09.007

[19] Feldmeier H . Head lice as vectors of pathogenic microorganisms. Trop Med Health 2023; 51:53. 37730694 10.1186/s41182-023-00545-5 PMC10510260

[20] Hammoud A , Louni M, Fenollar F, et al *Bartonella quintana* transmitted by head lice: an outbreak of trench fever in Senegal. Clin Infect Dis 2023; 76:1382–90. 36571112 10.1093/cid/ciac937

[21] Angelakis E , Diatta G, Abdisa A, et al Altitude-dependent *Bartonella quintana* genotype C in head lice, Ethiopia. Emerg Infect Dis 2011; 17:2357–9. 22172306 10.3201/eid1712.110453 PMC3311220

[22] Raoult D , Ndihokubwayo JB, Tissot-Dupont H, et al Outbreak of epidemic typhus associated with trench fever in Burundi. Lancet 1998; 352:353–8. 9717922 10.1016/s0140-6736(97)12433-3

[23] Pecoraro AJK , Pienaar C, Herbst PG, et al Causes of infective endocarditis in the Western Cape, South Africa: a prospective cohort study using a set protocol for organism detection and central decision making by an endocarditis team. BMJ Open 2021; 11:e053169. 10.1136/bmjopen-2021-053169 PMC8650472 34873007

[24] Barbe KP , Jaeggi E, Ninet B, et al *Bartonella quintana* endocarditis in a child. N Engl J Med 2000; 342:1841–2. 10866564 10.1056/NEJM200006153422418

[25] Thiam M , Fall PD, Gning SB, Grinda JM, Mainardi JL. *Bartonella quintana* infective endocarditis in an immunocompetent Senegalese man. Rev Med Interne 2002; 23:1036–7. 10.1016/s0248-8663(02)00734-8 12504245

[26] Mohammadian M , Butt S. Endocarditis caused by *Bartonella quintana*, a rare case in the United States. IDCases 2019; 17:e00533. 31384552 10.1016/j.idcr.2019.e00533 PMC6667705

[27] Marks F , Liu J, Soura AB, et al Pathogens that cause acute febrile illness among children and adolescents in Burkina Faso, Madagascar, and Sudan. Clin Infect Dis 2021; 73:1338–45. 33822011 10.1093/cid/ciab289 PMC8528393

[28] Mainardi JL , Drancourt M, Roland JM, et al *Bartonella* (*Rochalimaea*) *quintana* endocarditis in an Algerian farmer. Clin Microbiol Infect 1996; 1:275–6. 11866779 10.1016/s1198-743x(15)60288-9

[29] Znazen A , Rolain J-M, Hammami N, Kammoun S, Hammami A, Raoult D. High prevalence of *Bartonella quintana* endocarditis in Sfax, Tunisia. Am J Trop Med Hyg 2005; 72:503–7. 15891120

[30] Benslimani A , Fenollar F, Lepidi H, Raoult D. Bacterial zoonoses and infective endocarditis, Algeria. Emerg Infect Dis 2005; 11:216–24. 15752438 10.3201/eid1102.040668 PMC3320429

[31] Pecoraro AJ , Herbst PG, Doubell AF. Infective endocarditis in Africa: an urgent call for more data. Lancet Glob Heal 2022; 10:e8–9. 10.1016/S2214-109X(21)00489-7 34919860

[32] McCormick D , Rowan S, Pappert R, et al *Bartonella* seroreactivity among persons experiencing homelessness during an outbreak of *Bartonella quintana* in Denver, Colorado, 2020. Open Forum Infect Dis 2021; 8:ofab230. 34239947 10.1093/ofid/ofab230 PMC8135998

[33] Spach DH , Kanter AS, Dougherty MJ, et al *Bartonella* (*Rochalimaea*) *quintana* bacteremia in inner-city patients with chronic alcoholism. N Engl J Med 1995; 332:424–8. 7529895 10.1056/NEJM199502163320703

[34] Kostrzewski J . The epidemiology of trench fever. Bull Int Acad Pol Sci Let Cl Med 1949; 7:233–63. 24537054

[35] Gebremariam S , Moges T. Pediatric heart failure, lagging, and sagging of care in low income settings: a hospital based review of cases in Ethiopia. Cardiol Res Pract 2016; 2016:7147234. 27974990 10.1155/2016/7147234 PMC5128707

[36] WHO Strategic and Technical Advisory Group for Neglected Tropical Diseases. Recommendations for the adoption of additional diseases as neglected tropical diseases. 2017. Available at: https://cdn.who.int/media/docs/default-source/ntds/strategic-and-advisory-group-on-neglected-tropical-diseases-(stag-ntds)/tenth-ntdstag-report-2017.pdf?sfvrsn=9ec99065_2&download=truedate. Accessed 15 October 2023.

[37] Barbe KP , Jaeggi E, Ninet B, et al *Bartonella quintana* endocarditis in a child. N Engl J Med 2000; 342:1841–2. 10866564 10.1056/NEJM200006153422418

[38] Thiam M , Fall PD, Gning SB, Grinda JM, Mainardi JL. *Bartonella quintana* infective endocarditis in an immunocompetent Senegalese man. Rev Med Interne 2002; 23:1035–7. 12504245 10.1016/s0248-8663(02)00734-8

[39] Edouard S , Nabet C, Lepidi H, Fournier P-E, Raoult D. *Bartonella*, a common cause of endocarditis: a report on 106 cases and review. J Clin Microbiol 2015; 53:824–9. 25540398 10.1128/JCM.02827-14 PMC4390654

[40] Boodman C , Gupta N, Nelson C, van Griensven J. *Bartonella quintana* endocarditis: a systematic review of individual cases. Clin Infect Dis 2023. doi:10.1093/cid/ciad706 10.1093/cid/ciad706 37976173

[41] Karthikeyan K . Mass drug administration in neglected tropical diseases: beyond elimination. Lancet Glob Health 2023; 11:e813–4. 37202012 10.1016/S2214-109X(23)00213-9

[42] Jackson LA , Spach DH. Emergence of *Bartonella quintana* infection among homeless persons. Emerg Infect Dis 1996; 2:141–4. 8903217 10.3201/eid0202.960212 PMC2639836

[43] Maggi RG , Richardson T, Breitschwerdt EB, Miller JC. Development and validation of a droplet digital PCR assay for the detection and quantification of *Bartonella* species within human clinical samples. J Microbiol Methods 2020; 176:106022. 32795640 10.1016/j.mimet.2020.106022

[44] El Hamzaoui B , Laroche M, Almeras L, Bérenger J-M, Raoult D, Parola P. Detection of *Bartonella* spp in fleas by MALDI-TOF MS. PLoS Negl Trop Dis 2018; 12:e0006189. 29451890 10.1371/journal.pntd.0006189 PMC5833284

[45] El Hamzaoui B , Laroche M, Parola P. Detection of *Bartonella* spp in *Cimex lectularius* by MALDI-TOF MS. Comp Immunol Microbiol Infect Dis 2019; 64:130–7. 31174687 10.1016/j.cimid.2019.03.001

[46] Wolf LA , Cherry NA, Maggi RG, Breitschwerdt EB. In pursuit of a stealth pathogen: laboratory diagnosis of bartonellosis. Clin Microbiol Newsl 2014; 36:33–9.

[47] Agan BK , Dolan MJ. Laboratory diagnosis of *Bartonella* infections. Clin Lab Med 2002; 22:937–62. 12489289 10.1016/s0272-2712(02)00017-3

[48] Foucault C , Raoult D, Brouqui P. Randomized open trial of gentamicin and doxycycline for eradication of *Bartonella quintana* from blood in patients with chronic bacteremia. Antimicrob Agents Chemother 2003; 47:2204–7. 12821469 10.1128/AAC.47.7.2204-2207.2003 PMC161867

[49] Spach DH . *Bartonella quintana* infections: clinical features, diagnosis, and treatment. 2023. Available at: https://www.uptodate.com/contents/bartonella-quintana-infections-clinical-features-diagnosis-and-treatment. Accessed 16 October 2023.

[50] World Health Organization . WHO H5 reference laboratories. 2023. Available at: https://www.who.int/initiatives/global-influenza-surveillance-and-response-system/h5-reference-laboratoriesdate. Accessed 20 October 2023.

[51] Hart JT . The inverse care law. Lancet 1971; 297:405–12. 10.1016/s0140-6736(71)92410-x 4100731

