# Supplementary material for: Hidden Burden of Bartonella quintana on the African Continent: Should the Bacterial Infection Be Considered a Neglected Tropical Disease?
Source: Open Forum Infect Dis. 2023 Dec 27;11(2):ofad672. doi: 10.1093/ofid/ofad672 (PMC10873695; doi:10.1093/ofid/ofad672)
Supplement: ofad672_Supplementary_Data [file ofad672_supplementary_data.zip › Appendix 1 References map.docx]

**References for map of *B. quintana* infective endocarditis and louse positivity on the African continent**

1 Benslimani A, Fenollar F, Lepidi H, Raoult D. Bacterial zoonoses and infective endocarditis, Algeria. *Emerg Infect Dis* 2005; **11**: 216–24.

2 Berdagué P, Clavé D, Archambaud M, *et al.* Bartonella endocarditis on native valves: Two case reports . *Arch Mal Coeur Vaiss* 1998; **91**: 1277–81.

3 Boodman C, Wuerz T, Lagacé-Wiens P, *et al.* Serologic testing for &lt;em&gt;Bartonella&lt;/em&gt; in Manitoba, Canada, 2010–2020: a retrospective case series. *C Open* 2022; **10**: E476 LP-E482.

4 Boudebouch N, Sarih M, Chakib A, *et al.* Blood culture-negative endocarditis, Morocco. *Emerg Infect Dis* 2017; **23**: 1908–9.

5 García-Álvarez L, García-García C, Muñoz P, *et al.* Bartonella Endocarditis in Spain: Case Reports of 21 Cases. Pathog. . 2022; **11**. DOI:10.3390/pathogens11050561.

6 Goldstein LH, Saliba WR, Elias M, Zlotnik A, Raz R, Giladi M. Bartonella quintana endocarditis in east Africa. *Eur J Intern Med* 2005; **16**: 518–9.

7 Hipp S, von der Emde W, Kulke C, *et al.* Recurrent intracerebral haemorrhage in a 24-year-old female patient . *Internist* 2022; **63**: 103–9.

8 Kularatne R. Bartonella endocarditis and a diagnostic algorithm for culture-negative endocarditis in South Africa. *South African J Epidemiol Infect* 2015; **30**: 150.

9 Mainardi JL, Drancourt M, Roland JM, *et al.* Bartonella (Rochalimaea) quintana endocarditis in an Algerian farmer. *Clin Microbiol Infect* 1996; **1**: 275–6.

10 Mohammadian M, Butt S. Endocarditis caused by Bartonella Quintana, a rare case in the United States. *IDCases* 2019; **17**: e00533–e00533.

11 Montcriol A, Benard F, Fenollar F, *et al.* Fatal myocarditis-associated Bartonella quintana endocarditis: A case report. *J Med Case Rep* 2009; **3**. DOI:10.4076/1752-1947-3-7325.

12 Moodley VM, Zeeman MTS, van Greune CHJ, Corcoran C. Culture-negative endocarditis due to Bartonella quintana. *South African Med J* 2016; **106**: 470–1.

13 Pecoraro AJK, Pienaar C, Herbst PG, *et al.* Causes of infective endocarditis in the Western Cape, South Africa: a prospective cohort study using a set protocol for organism detection and central decision making by an endocarditis team. *BMJ Open* 2021; **11**: e053169.

14 Plantinga NL, Vos RJ, Georgieva L, Roescher N. Bartonella quintana as a cause for prosthetic valve endocarditis and post-sternotomy mediastinitis. *Access Microbiol* 2021; **3**. DOI:10.1099/acmi.0.000217.

15 Barbe KP, Jaeggi E, Ninet B, *et al.* Bartonella quintana Endocarditis in a Child. *N Engl J Med* 2000; **342**: 1841–2.

16 Raoult D, PE F, Drancourt M, al et. DIagnosis of 22 new cases of bartonella endocarditis. *Ann Intern Med* 1996; **125**: 646–52.

17 Robert M, Lecomte R, Michel M, *et al.* Bartonella quintana infective endocarditis in a homeless man with unexpected positive blood culture. *IDCases* 2022; **30**: e01647.

18 Sondermeijer HP, Claas ECJ, Orendi JM, Tamsma JT. Bartonella quintana prosthetic valve endocarditis detected by blood culture incubation beyond 10 days. *Eur J Intern Med* 2006; **17**: 441–3.

19 Tasher D, Raucher-Sternfeld A, Tamir A, Giladi M, Somekh E. Bartonella quintana, an Unrecognized Cause of Infective Endocarditis in Children in Ethiopia. *Emerg Infect Dis* 2017; **23**: 1246–52.

20 Thiam M, Fall PD, Gning SB, Grinda JM, Mainardi JL. Bartonella quintana infective endocarditis. In an immunocompetent senegalese man [3] . *Rev Med Interne* 2002; **23**: 1036–7.

21 Znazen A, Rolain J-M, Hammami N, Kammoun S, Hammami A, Raoult D. High prevalence of Bartonella quintana endocarditis in Sfax, Tunisia. *Am J Trop Med Hyg* 2005; **72**: 503–7.

22 Ouarti B, Mbogning Fonkou DM, Houhamdi L, Mediannikov O, Parola P. Lice and lice-borne diseases in humans in Africa: A narrative review. *Acta Trop* 2023; **237**: 106709.

23 Louni M, Amanzougaghene N, Mana N, *et al.* Detection of bacterial pathogens in clade E head lice collected from Niger’s refugees in Algeria. *Parasit Vectors* 2018; **11**. DOI:10.1186/s13071-018-2930-5.
